# Supplementary material for: Common synaptic phenotypes arising from diverse mutations in the human NMDA receptor subunit GluN2A
Source: Commun Biol. 2022 Feb 28;5:174. doi: 10.1038/s42003-022-03115-3 (PMC8885697; doi:10.1038/s42003-022-03115-3)
Supplement: Supplementary file 2 — Supplementary Information [file 42003_2022_3115_MOESM2_ESM.pdf]

Supplemental Information

Table of Contents

Supplemental Information *1*

Figures S1.1 – S1.11: Summary of Linear Mixed Models (LMM) **2**

Figure S1.1. NMDA-EPSC peak amplitudes in GluN2A/B double KO neurons rescued with GluN2A mutants 3

Figure S1.2. NMDA-EPSC decays in GluN2A/B double KO neurons rescued with GluN2A GOF mutants 4

Figure S1.3. Synaptic GFP-GluN1 expression rescued by expression of GluN2A mutants. 5

Figure S1.4. NMDA-EPSC peak amplitudes in GluN2A knockout neurons rescued with GluN2A mutants. 6

Figure S1.5. NMDA-EPSC decays in GluN2A knockout neurons rescued with GluN2A mutants. 7

Figure S1.6. NMDA-EPSC charge transfer in GluN2A knockout neurons rescued with GluN2A mutants. 8

Figure S1.7. AMPA-EPSC peak amplitudes in GluN2A knockout neurons rescued with GluN2A mutants. 9

Figure S1.8. NMDA-EPSC charge transfer in neurons expressing GluN2A knockout alleles. 10

Figure S1.9. NMDA-EPSC peak amplitudes in neurons expressing GluN2A knockout alleles. 11

Figure S1.10. NMDA-EPSC decays constant in neurons expressing GluN2A knockout alleles. 12

Figure S1.11. NMDA-EPSC risetimes in neurons expressing GluN2A knockout alleles. 13

Figure S2: Likely toxicity from overexpressing the GluN2A L812M mutant **14**

Figure S3: Effective rescue of native mouse GluN2A by human GluN2A **15**

Figure S4: GluN2A mutations are not associated with large effects on AMPAR-EPSCs. **16**

Figure S5: Structural models of the wildtype and mutated amino acids for LOF mutations **17**

Table S1. Matrix of orthogonal contrasts based on *a priori* clustering of mutations. **18**

Table S2: Clinical features of patients reported previously with *GRIN2A* mutations used in this study. **19**

Table S3: Primers used to generate mutant GluN2A constructs from WT pCI-Neo *GRIN2A* **20**

Table S4. Experiment sample sizes **21**

Supplemental References **22**

Figures S1.1 – S1.11: Summary of Linear Mixed Models (LMM)

The top of each of the following figures includes: the `lmer` (Wilkinson-Rogers-Pinheiro-Bates) model formula, an ANOVA table with *F* (*df*, df_{res}) statistics, *p*-values, and Bayes factors for hypothesis tests of interests on fixed effects, and a table of group sample size and intraclass correlation (ICC) for nuisance random effects (and the residual, unexplained error). The bottom of each of the following figures (1.1-1.11) includes a series of plots for assessing the validity of assumptions made by the LMM: **a**) Standardized residuals plotted against the fitted values with smoothed conditional mean (red) and conditional median accompanied by lower and upper quartiles (blue) ; **b**) Histogram of residual overlaid with fitted Gaussian distribution (red) and kernel density estimate (blue); **c**) quantile-quantile (Q-Q) plot with 95% confidence bands; and **d**) Stem-and-leaf plot of Cook's distance.

Figure S1.1. NMDA-EPSC peak amplitudes in GluN2A/B double KO neurons rescued with GluN2A mutants

Top. Table summary of a Linear Mixed Model (LMM) predicting NMDA-EPSC peak amplitude from the co-transfection of *GRIN2A* mutants and Cre-GFP into CA1 neurons of *grin2a*$^{fl/fl}$*2b*$^{fl/fl}$ neurons. Fixed effects are summarised as an ANOVA table with the interaction term decomposed into orthogonal contrasts (A-E, see Table S1). The *p*-value and Bayes factor for the interaction term omnibus test indicate a highly significant effect of transfecting GluN2A mutants on the peak amplitude of NMDA-EPSCs in GluN2A/B double knockout (KO) neurons. Of the variance not accounted for by the fixed effects, 41% and 59% was explained by variability between recording pairs or residual (unexplained) variance respectively. ***Bottom***. Model residuals appeared to be homoscedastic (**a**) and normally distributed (**b**, **c**), without outliers (**a, c**) or overly influential data points (**d**). A breakdown of samples sizes is reported in Table S4.

**Response**					
NMDA-EPSC peak amplitude (peak)					
**Formula**					
$\log(\boldsymbol{peak}) \sim mutation * transfection + (1\vert animal/slice/pair)$					
**Source (Fixed)**	***F***	***df***	***df$_{res}$***	***p***	***BF$_{10}$***
mutation	7.89	5	8.8		
transfection	433.89	1	87		
mutation:transfection	11.35	5	87	<.001	4.14E+06
A. WT *vs.* K669N, L812M, C436R, T531M, R518H	31.22	1	87	<.001	
B. K669N, L812M *vs.* C436R, T531M, R518H	25.94	1	87	<.001	
C. R518H *vs.* C436R, T531M	0.01	1	87	.93	
D. C436R *vs.* T531M	0.14	1	87	.71	
E. K669N *vs.* L812M	0.00	1	87	.98	
**Source (random)**	***N***	**ICC**			
pair:(slice:animal)	93	.41			
slice:animal	54	.00			
animal	16	.00			
Residual		.59			

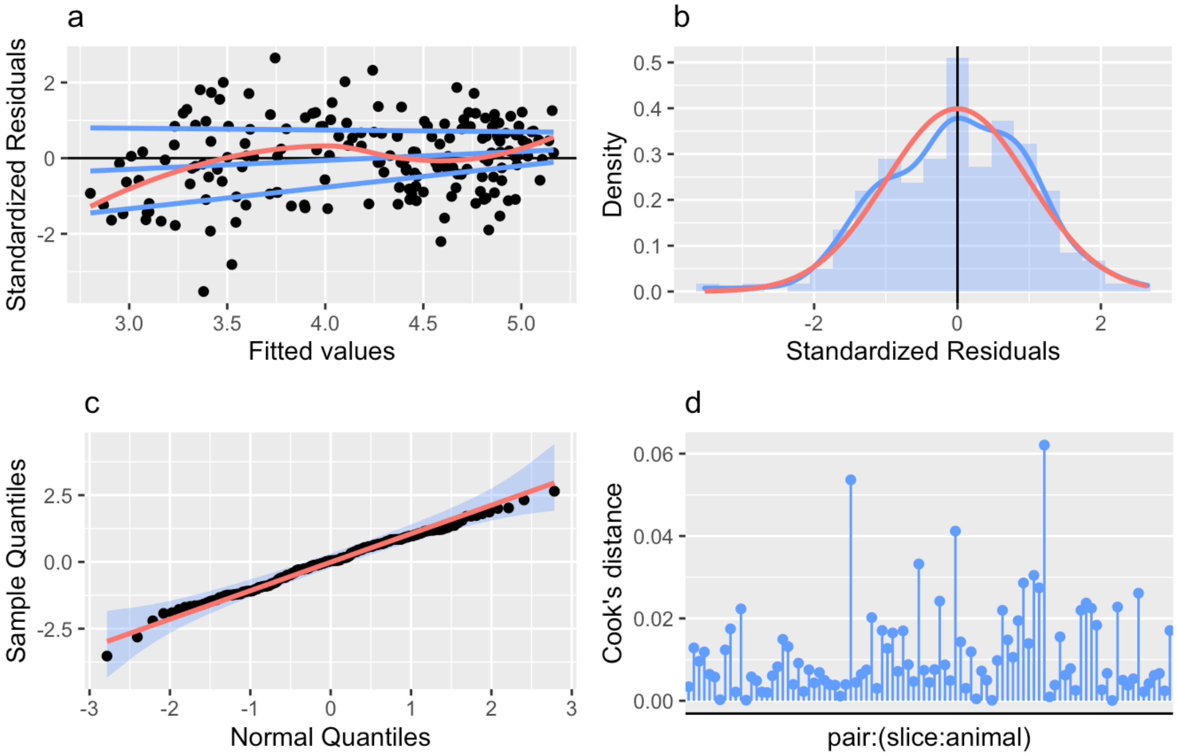

Figure S1.2. NMDA-EPSC decays in GluN2A/B double KO neurons rescued with GluN2A GOF mutants

Top. Table summary of a LMM predicting NMDA-EPSC decay time constant from the co-transfection of *GRIN2A* mutants and Cre-GFP in CA1 neurons of *grin2a*$^{fl/fl}$*2b*$^{fl/fl}$ neurons. Fixed effects are summarised as an ANOVA table with the interaction followed up with posthoc pairwise comparisons. The *p*-value and Bayes factor for the interaction term omnibus test indicate a highly significant effect of transfecting GluN2A mutants on the decay time constant of NMDA-EPSCs in GluN2A/B double knockout (KO) neurons. Of the variance not accounted for by the fixed effects, 20%, 7%, 27% and 46% was explained by variability between recording pairs, slices, animals and by residual (unexplained) variance respectively. ***Bottom***. Model residuals appeared to be homoscedastic (**a**) and normally distributed (**b**, **c**), without overt outliers (**a, c**) or overly influential data points (**d**). A breakdown of samples sizes is reported in Table S4.

**Response**					
NMDA-EPSC decay time constant (decay)					
**Formula**					
$\log(\boldsymbol{decay}) \sim mutation * transfection + (1 \mid animal/slice/pair)$					
**Source (Fixed)**	***F***	***df***	***df_{res}***	***p***	***BF_{10}***
mutation	69.01	2	5.71		
transfection	19.06	1	49		
mutation:transfection	190.3	2	49	<.001	7.61E+40
**Posthoc test**	***t***		***df***	***p_{adj}***	
WT *vs.* K669N	5.3		49	<.001	
WT *vs.* L812M	13.29		49	<.001	
K669N *vs.* L812M	9.1		49	<.001	
**Source (random)**	***N***	**ICC**			
pair:(slice:animal)	52	.20			
slice:animal	29	.07			
animal	9	.27			
Residual		.46			

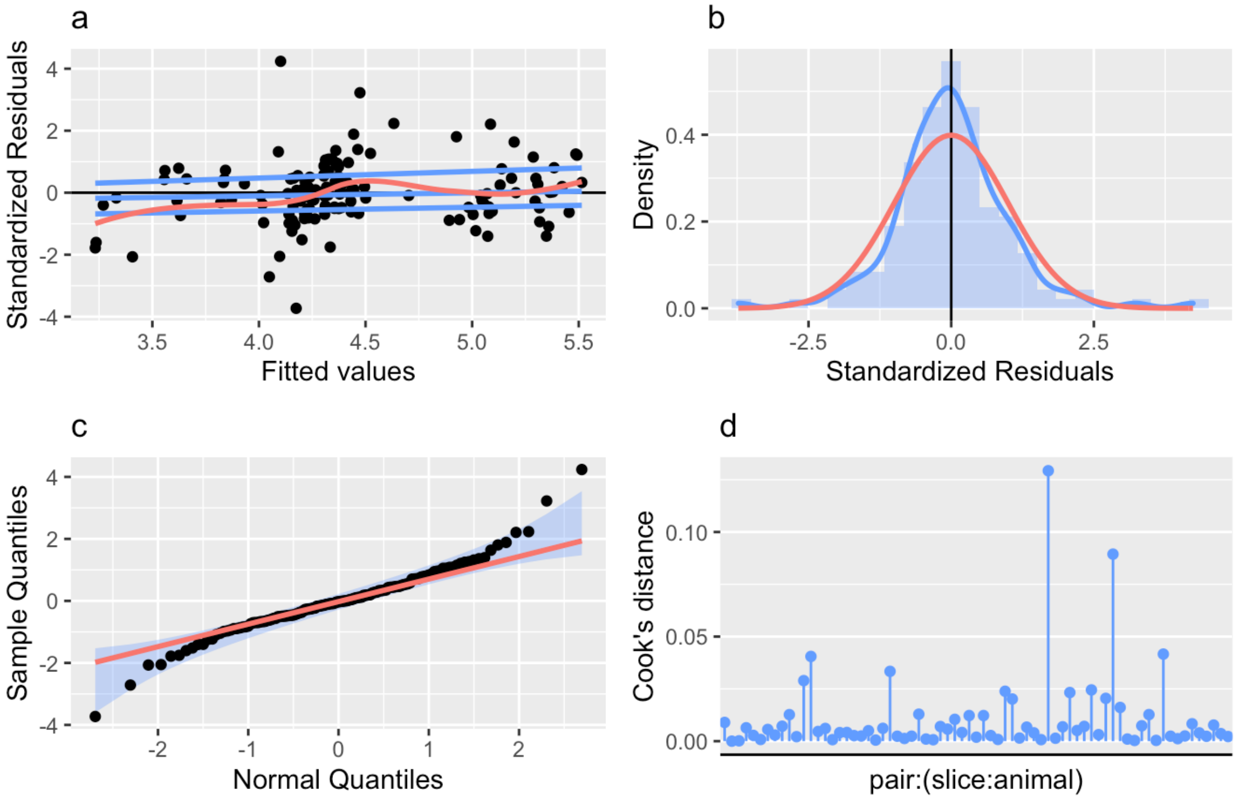

Figure S1.3. Synaptic GFP-GluN1 expression rescued by expression of GluN2A mutants.

Top. Table summary of a LMM predicting fluorescence intensity from GFP-GluN1 and Homer1c expressed in cultured hippocampal neurons co-transfected with different *GRIN2A* mutants. Fixed effects are summarised as an ANOVA table with the interaction term decomposed into orthogonal contrasts (A-E, see Table S1). The *p*-value and Bayes factor for the interaction term omnibus test indicate a highly significant effect of GluN2A mutants on the fluorescence of GFP-GluN1 relative to Homer1c-tdTomato. Of the variance not accounted for by the fixed effects, 19, 9 and 72% was explained by variability between regions of interest (roi), experiments or residual (unexplained) variance respectively. ***Bottom***. Model residuals appeared to be mostly homoscedastic (**a**) and normally distributed (**b**, **c**), without overt outliers (**a, c**) or overly influential data points (**d**). A breakdown of samples sizes is reported in Table S4.

**Response**					
Fluorescence intensity (fluorescence)					
**Formula**					
$\log(\boldsymbol{fluorescence}) \sim mutation * protein + (1 \mid expt/roi)$					
**Source (Fixed)**	***F***	***df***	df_{res}	***p***	BF_{10}
mutation	21.02	5	126		
protein	1902	1	185		
mutation:protein	10.29	5	185	<.001	4.82E+04
A. WT *vs*. K669N, L812M, C436R, T531M, R518H	12.71	1	185	<.001	
B. K669N, L812M *vs.* C436R, T531M, R518H	6.9	1	185	.009	
C. R518H *vs.* C436R, T531M	21.65	1	185	<.001	
D. C436R *vs.* T531M	7.45	1	185	.007	
E. K669N *vs.* L812M	9.32	1	185	.003	
**Source (random)**	***N***	**ICC**			
roi:expt	191	.19			
expt	8	.09			
Residual		.72			

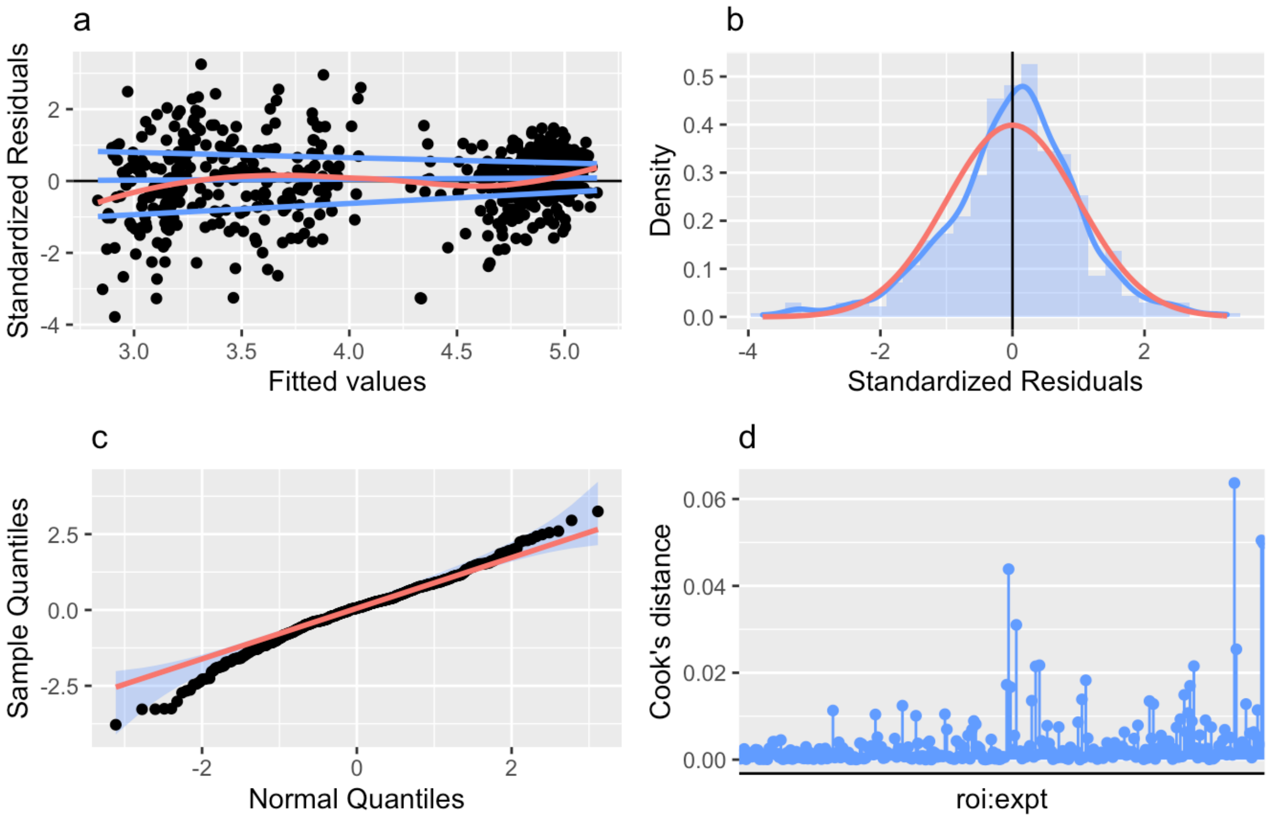

Figure S1.4. NMDA-EPSC peak amplitudes in GluN2A knockout neurons rescued with GluN2A mutants.

Top. Table summary of a LMM predicting NMDA-EPSC peak amplitude from the co-transfection of *GRIN2A* mutants and Cre-GFP in CA1 neurons of *grin2a*$^{fl/fl}$ neurons. Fixed effects are summarised as an ANOVA table with the interaction term decomposed into orthogonal contrasts (A-E, see Table S1). The Bayes factor for the interaction term omnibus test provides evidence more in favour of no effect of GluN2A mutants on the peak amplitude of NMDA-EPSCs in GluN2A knockout (KO) neurons. Of the variance not accounted for by the fixed effects, 24%, 22%, 37% and 16% was explained by variability between recording pairs, slices, animals, and residual (unexplained) variance respectively. ***Bottom***. Model residuals appeared to be homoscedastic (**a**) and normally distributed (**b**, **c**), without outliers (**a, c**) or influential data points (**d**). A breakdown of samples sizes is reported in Table S4.

**Response**					
NMDA-EPSC peak amplitude (peak)					
**Formula**					
$\log(\boldsymbol{peak}) \sim mutation * transfection + (1\|animal/slice/pair)$					
**Source (Fixed)**	***F***	***df***	***df***$_{res}$	***p***	***BF***$_{10}$
mutation	0.72	5	19.8		
transfection	78.02	1	119		
mutation:transfection	0.87	5	119	.50	0.0838
A. WT vs K669N, L812M, C436R, T531M, R518H	2.47	1	119	.12	
B. K669N, L812M vs. C436R, T531M, R518H	1.88	1	119	.17	
C. R518H vs. C436R, T531M	0.25	1	119	.62	
D. C436R vs. T531M	0.00	1	119	.99	
E. K669N vs. L812M	0.14	1	119	.71	
**Source (random)**	***N***	**ICC**			
pair:(slice:animal)	125	.24			
slice:animal	78	.22			
animal	27	.37			
Residual		.16			

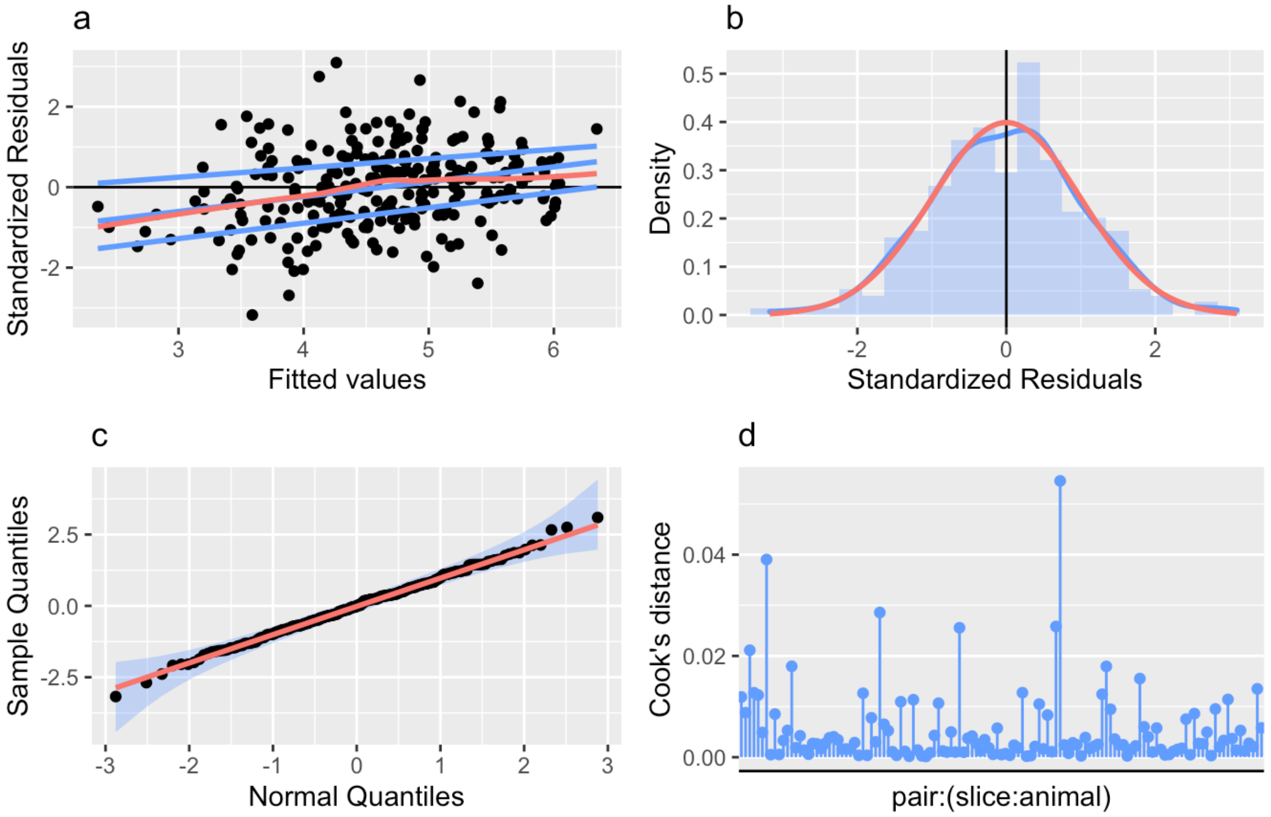

Figure S1.5. NMDA-EPSC decays in GluN2A knockout neurons rescued with GluN2A mutants.

Top. Table summary of a LMM predicting NMDA-EPSC decay time constant from the co-transfection of *GRIN2A* mutants and Cre-GFP in CA1 neurons of $grin2a^{fl/fl}$ neurons. Fixed effects are summarised as an ANOVA table with the interaction term decomposed into orthogonal contrasts (A-E, see Table S1). The *p*-value and Bayes factor for the interaction term omnibus test indicate a highly significant effect of transfecting GluN2A mutants on the decay time constant of NMDA-EPSCs in GluN2A knockout (KO) neurons. Of the variance not accounted for by the fixed effects, 4%, 12%, 16% and 68% was explained by variability between recording pairs, slices, animals, and residual (unexplained) variance respectively. ***Bottom***. Model residuals appeared to be homoscedastic (**a**) and normally distributed (**b**, **c**), without outliers (**a, c**) or influential data points (**d**). A breakdown of samples sizes is reported in Table S4.

**Response**					
NMDA-EPSC decay time constant (decay)					
**Formula**					
$\log(\boldsymbol{decay}) \sim mutation * transfection + (1\vert animal/slice/pair)$					
**Source (Fixed)**	***F***	***df***	df_{res}	***p***	BF_{10}
mutation	3.06	5	19.3		
transfection	179.6	1	119		
mutation:transfection	12.09	5	119	<.001	3.43E+08
A. WT vs K669N, L812M, C436R, T531M, R518H	37.09	1	119	<.001	
B. K669N, L812M vs. C436R, T531M, R518H	28.34	1	119	<.001	
C. R518H vs. C436R, T531M	0.13	1	119	.72	
D. C436R vs. T531M	0.23	1	119	.63	
E. K669N vs. L812M	0.12	1	119	.73	
**Source (random)**	***N***	**ICC**			
pair:(slice:animal)	125	.04			
slice:animal	78	.12			
animal	27	.16			
Residual		.68			

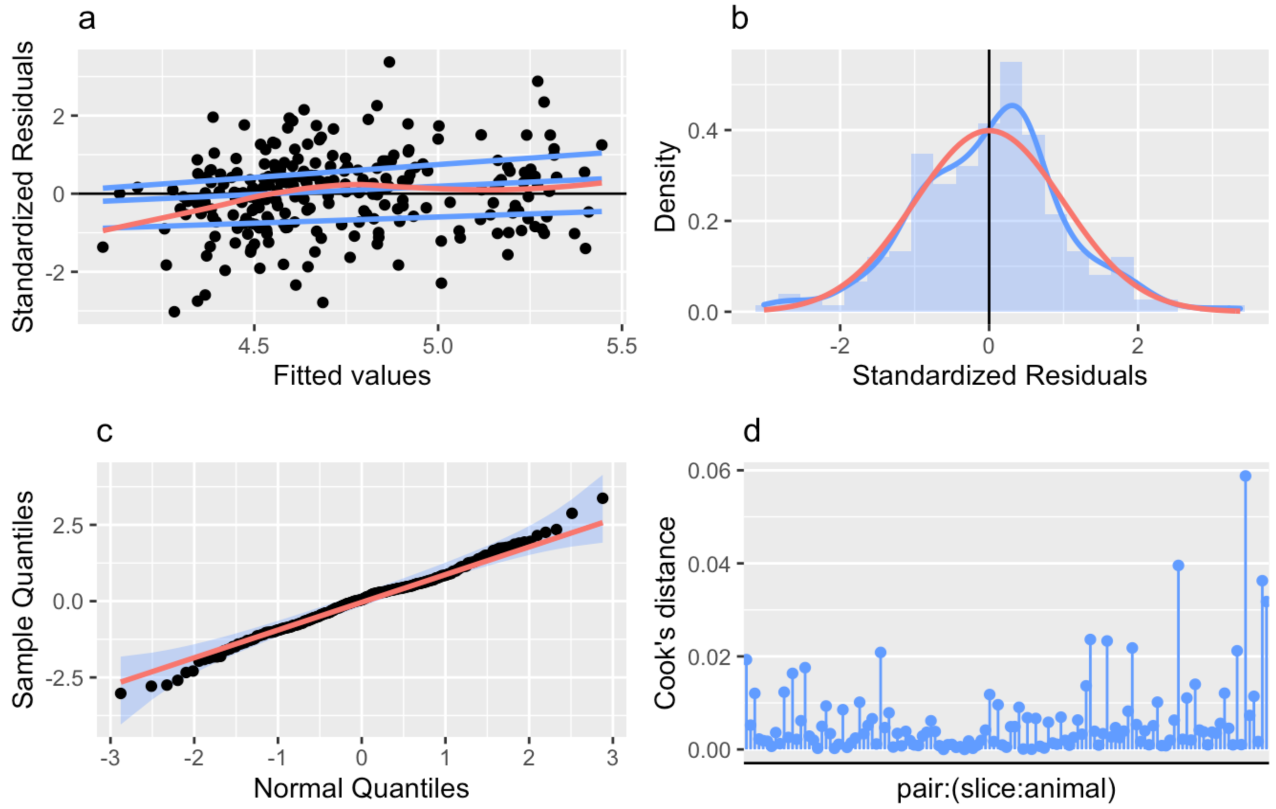

Figure S1.6. NMDA-EPSC charge transfer in GluN2A knockout neurons rescued with GluN2A mutants.

Top. Table summary of a LMM predicting NMDA-EPSC charge transfer from the co-transfection of *GRIN2A* mutants and Cre-GFP in CA1 neurons of *grin2a*$^{fl/fl}$ neurons. Fixed effects are summarised as an ANOVA table with the interaction term decomposed into orthogonal contrasts (A-E, see Table S1). The Bayes factor for the interaction term omnibus test provides weak evidence for no effect of GluN2A mutants on the charge transfer of NMDA-EPSCs in GluN2A knockout (KO) neurons. Of the variance not accounted for by the fixed effects, 33%, 22%, 27% and 18% was explained by variability between recording pairs, slices, animals, and residual (unexplained) variance respectively. ***Bottom***. Model residuals appeared to be homoscedastic (**a**) and normally distributed (**b**, **c**), without outliers (**a, c**) or influential data points (**d**). A breakdown of samples sizes is reported in Table S4.

**Response**					
NMDA-EPSC charge transfer (charge)					
**Formula**					
$\log(\boldsymbol{charge}) \sim mutation * transfection + (1|animal/slice/pair)$					
**Source (Fixed)**	***F***	***df***	***df$_{res}$***	***p***	***BF$_{10}$***
mutation	1.05	5	19.5		
transfection	0	1	119		
mutation:transfection	2.13	5	119	.07	0.632
A. WT vs K669N, L812M, C436R, T531M, R518H	4.18	1	119	.04	
B. K669N, L812M vs. C436R, T531M, R518H	4.26	1	119	.04	
C. R518H vs. C436R, T531M	2.47	1	119	.12	
D. C436R vs. T531M	0.17	1	119	.68	
E. K669N vs. L812M	0.32	1	119	.57	
**Source (random)**	***N***	**ICC**			
pair:(slice:animal)	125	.33			
slice:animal	78	.22			
animal	27	.27			
Residual		.18			

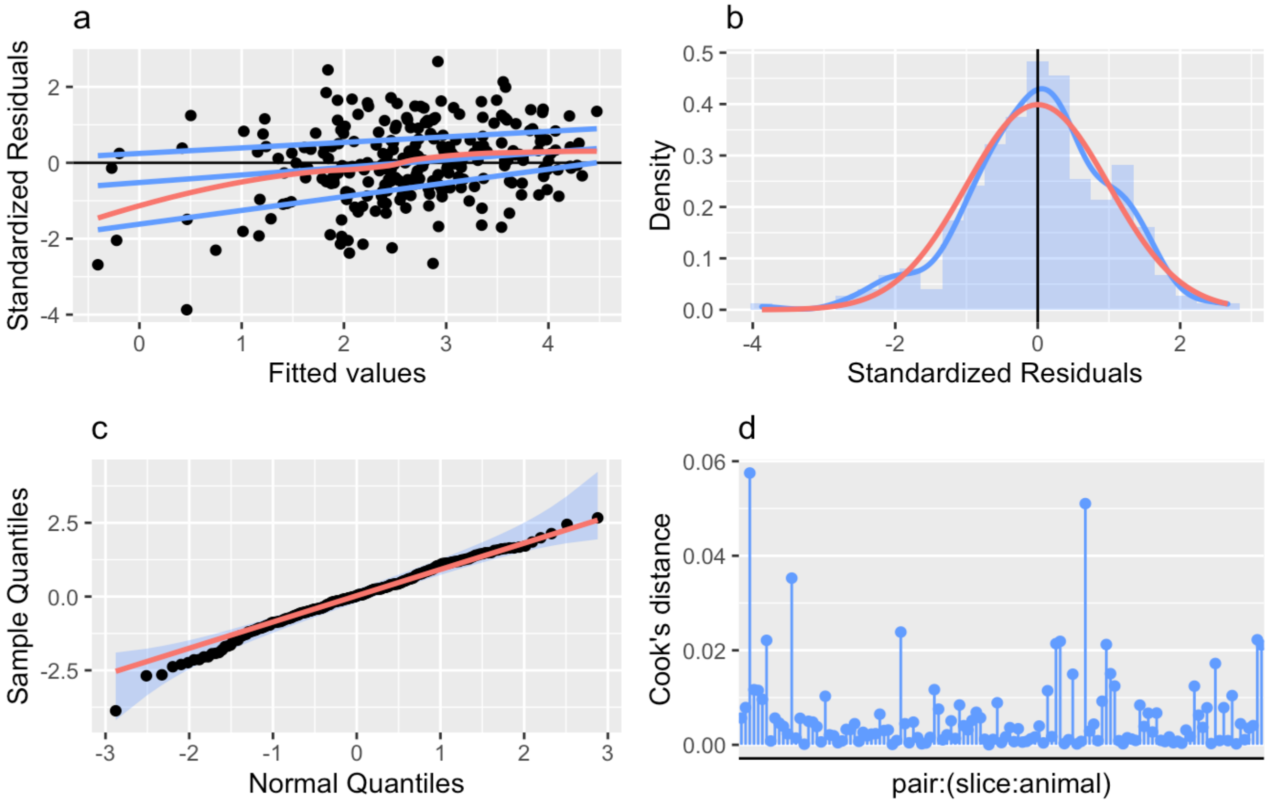

Figure S1.7. AMPA-EPSC peak amplitudes in GluN2A knockout neurons rescued with GluN2A mutants.

Top. Table summary of a LMM predicting AMPA-EPSC peak amplitude from the co-transfection of *GRIN2A* mutants and Cre-GFP in CA1 neurons of *grin2a*$^{fl/fl}$ neurons. Fixed effects are summarised as an ANOVA table with the interaction term decomposed into orthogonal contrasts (A-E, see Table S1). The Bayes factor for the interaction term omnibus test provides reasonable evidence for no effect of GluN2A mutants on the peak amplitude of AMPA-EPSCs in GluN2A knockout (KO) neurons. Of the variance not accounted for by the fixed effects, 27%, 40% and 33% was explained by variability between recording pairs, animals, and residual (unexplained) variance respectively. ***Bottom***. Model residuals appeared to be homoscedastic (**a**) and normally distributed (**b**, **c**), without outliers (**a, c**) or influential data points (**d**). A breakdown of samples sizes is reported in Table S4.

**Response**					
AMPA-EPSC peak amplitude (peak)					
**Formula**					
$\log(\boldsymbol{peak}) \sim mutation * transfection + (1\|animal/slice/pair)$					
**Source (Fixed)**	***F***	***df***	***df$_{res}$***	***p***	***BF$_{10}$***
mutation	2.66	5	19.95		
transfection	2.38	1	119		
mutation:transfection	1.49	5	119	.20	0.267
A. WT vs K669N, L812M, C436R, T531M, R518H	0.04	1	119	.84	
B. K669N, L812M vs. C436R, T531M, R518H	7.23	1	119	.008	
C. R518H vs. C436R, T531M	0.15	1	119	.70	
D. C436R vs. T531M	0.05	1	119	.82	
E. K669N vs. L812M	0.01	1	119	.93	
**Source (random)**	***N***	**ICC**			
pair:(slice:animal)	125	.27			
slice:animal	78	.00			
animal	27	.40			
Residual		.33			

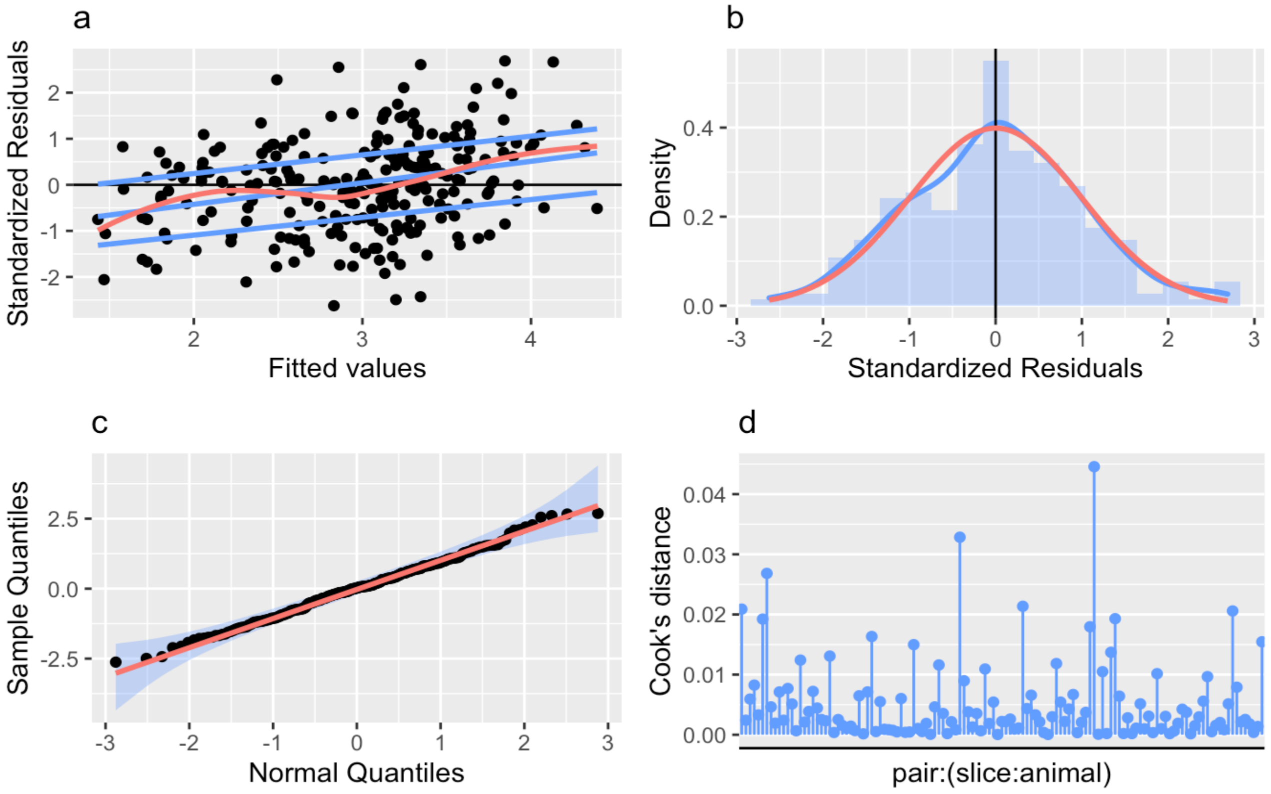

Figure S1.8. NMDA-EPSC charge transfer in neurons expressing GluN2A knockout alleles.

Top. Table summary of a LMM predicting NMDA-EPSC charge transfer from the transfection of Cre-GFP in CA1 neurons of *grin2a*$^{fl/fl}$ neurons. Fixed effects are summarised as an ANOVA table with the interaction term split into polynomial contrasts and followed up with posthoc pairwise comparisons. The Bayes factor for the interaction term omnibus test provides reasonable evidence for no effect of loss-of-function (null) GluN2A alleles on the charge transfer of NMDA-EPSCs. Of the variance not accounted for by the fixed effects, 1%, 32%, 31% and 36% was explained by variability between recording pairs, slices, animals, and residual (unexplained) variance respectively. ***Bottom***. Model residuals appeared to be homoscedastic (**a**) and normally distributed (**b**, **c**), without outliers (**a**, **c**) or influential data points (**d**). A breakdown of samples sizes is reported in Table S4.

**Response**					
NMDA-EPSC charge transfer (charge)					
**Formula**					
$\log(\boldsymbol{charge}) \sim genotype * transfection + (1 \mid animal/slice/pair)$					
**Source (Fixed)**	***F***	***df***	***df$_{res}$***	***p***	***BF$_{10}$***
genotype	0.09	2	10.09		
transfection	0.01	1	96		
genotype:transfection	1.21	2	96	.30	0.277
Linear	2.39	1	96	.13	
Quadratic	0.01	1	96	.91	
**Source (random)**	***N***	**ICC**			
pair:(slice:animal)	125	.01			
slice:animal	78	.32			
animal	27	.31			
Residual		.36			

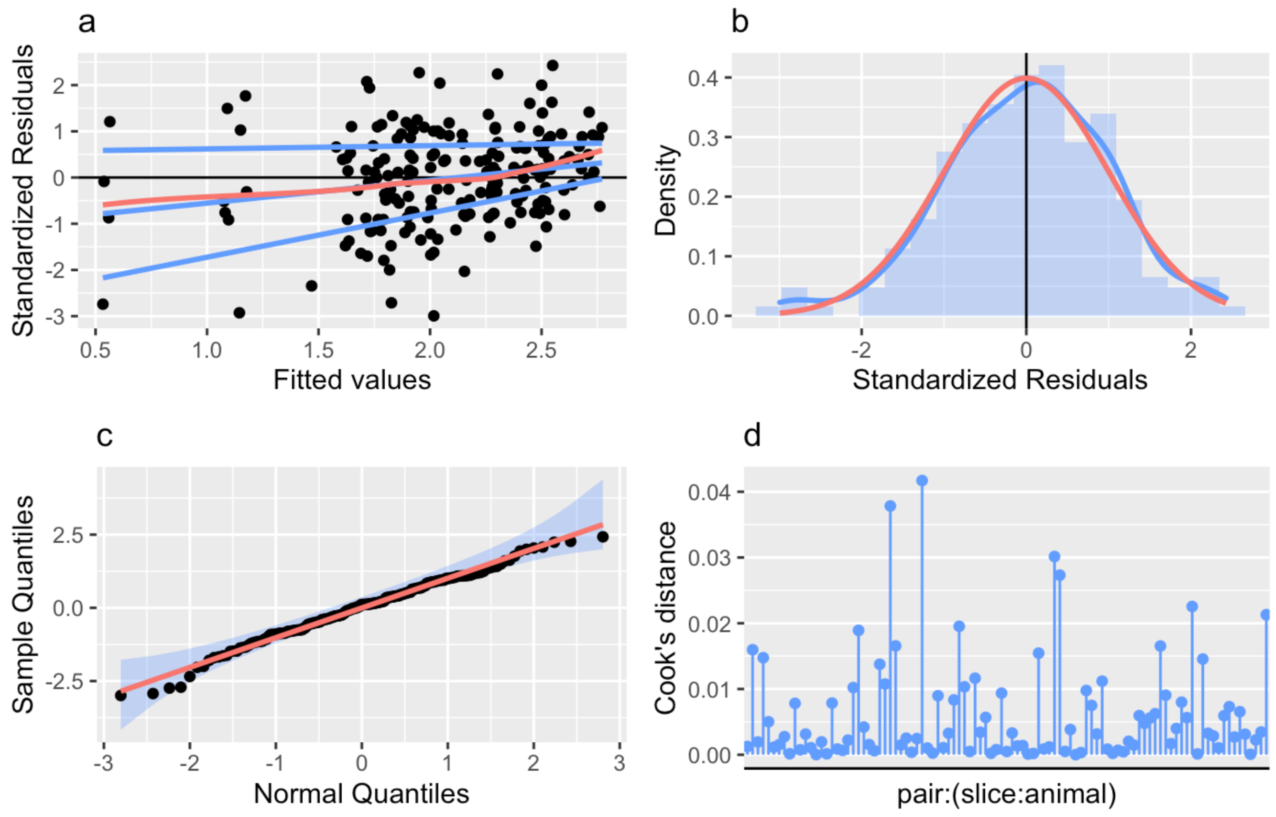

Figure S1.9. NMDA-EPSC peak amplitudes in neurons expressing GluN2A knockout alleles.

Top. Table summary of a LMM predicting NMDA-EPSC peak amplitude from the transfection of Cre-GFP in CA1 neurons of *grin2a*$^{fl/fl}$ neurons. Fixed effects are summarised as an ANOVA table with the interaction term split into polynomial contrasts and followed up with posthoc pairwise comparisons. The *p*-value and Bayes factor for the interaction term omnibus test indicate a highly significant effect of loss-of-function (null) GluN2A alleles on the peak amplitude of NMDA-EPSCs. Of the variance not accounted for by the fixed effects, 27%, 39% and 44% was explained by variability between slices, animals, and residual (unexplained) variance respectively. ***Bottom***. Model residuals appeared to be homoscedastic (**a**) and normally distributed (**b**, **c**), without outliers (**a, c**) or influential data points (**d**). A breakdown of samples sizes is reported in Table S4.

**Response**					
NMDA-EPSC peak amplitude (peak)					
**Formula**					
$\log(\boldsymbol{peak}) \sim genotype * transfection + (1\vert animal/slice/pair)$					
**Source (Fixed)**	***F***	***df***	***df$_{res}$***	***p***	***BF$_{10}$***
genotype	0.83	2	10.07		
transfection	25.46	1	96		
genotype:transfection	8.61	2	96	<.001	227
Linear	13	1	96	<.001	
Quadratic	3.76	1	96	.06	
**Posthoc test**	***t***		***df***	***p$_{adj}$***	
+/+ vs. +/-	-0.01		96	.99	
+/+ vs. -/-	-3.61		96	.001	
+/- vs. -/-	-3.47		96	.001	
**Source (random)**	***N***	***ICC***			
pair:(slice:animal)	99	.00			
slice:animal	53	.27			
animal	14	.29			
Residual		.44			

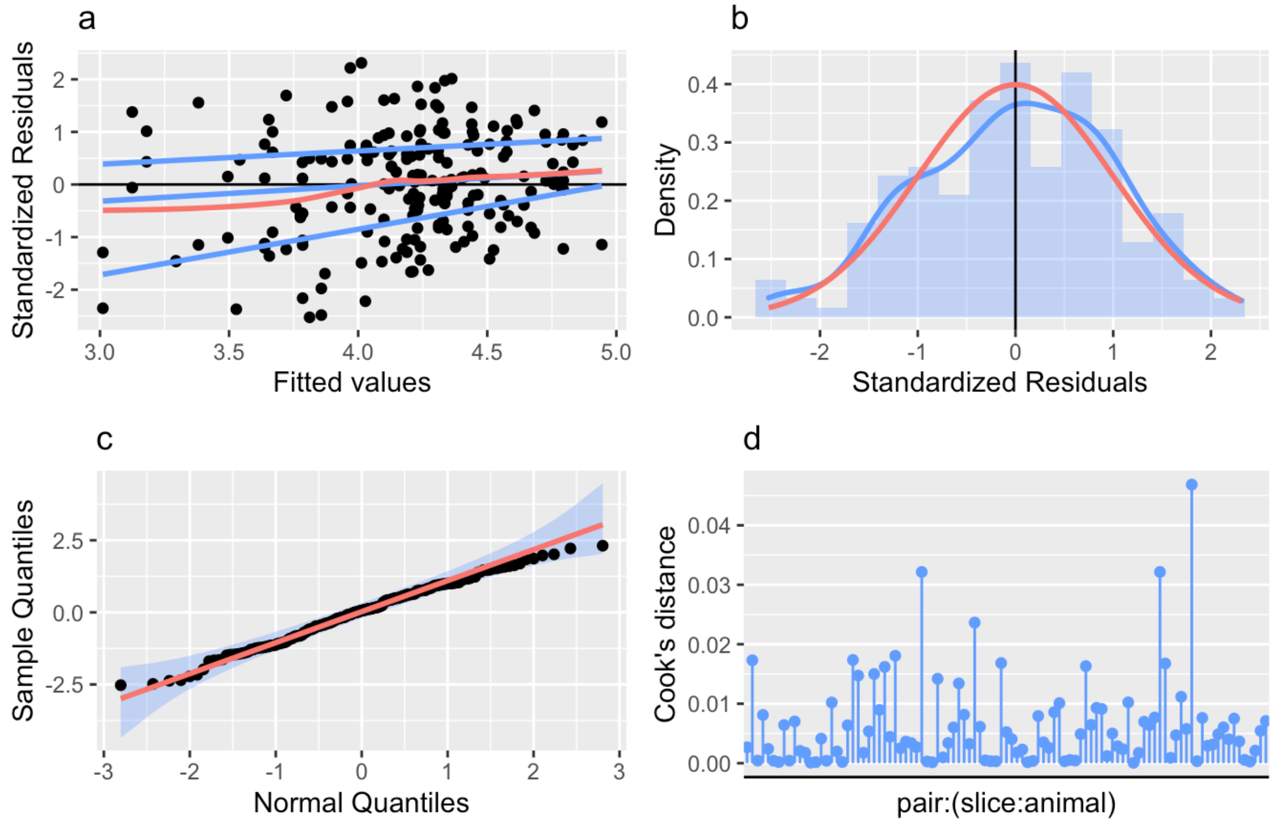

Figure S1.10. NMDA-EPSC decays constant in neurons expressing GluN2A knockout alleles.

Top. Table summary of a LMM predicting NMDA-EPSC decay time constant amplitude from the transfection of Cre-GFP in CA1 neurons of *grin2a*$^{fl/fl}$ neurons. Fixed effects are summarised as an ANOVA table with the interaction term split into polynomial contrasts and followed up with posthoc pairwise comparisons. The *p*-value and Bayes factor for the interaction term omnibus test indicate a highly significant effect of loss-of-function (null) GluN2A alleles on the decay time constant of NMDA-EPSCs. Of the variance not accounted for by the fixed effects, 14%, 26%, 25% and 35% was explained by variability between recording pairs, slices, animals, and residual (unexplained) variance respectively. ***Bottom***. Model residuals appeared to be homoscedastic (**a**) and normally distributed (**b**, **c**), without outliers (**a, c**) or influential data points (**d**). A breakdown of samples sizes is reported in Table S4.

**Response**					
NMDA-EPSC decay time constant (decay)					
**Formula**					
$\log(\boldsymbol{decay}) \sim genotype * transfection + (1 \vert animal/slice/pair)$					
**Source (Fixed)**	***F***	***df***	df_{res}	***p***	BF_{10}
genotype	4.91	2	9.90		
transfection	156.8	1	96		
genotype:transfection	76.23	2	96	<.001	4.67E+19
Linear	140.1	1	96	<.001	
Quadratic	9.9	1	96	.002	
**Posthoc test**	***t***		***df***	p_{adj}	
+/+ vs. +/-	2.84		96	.006	
+/+ vs. -/-	11.84		96	<.001	
+/- vs. -/-	8.52		96	<.001	
**Source (random)**	***N***	**ICC**			
pair:(slice:animal)	125	.14			
slice:animal	78	.26			
animal	27	.25			
Residual		.35			

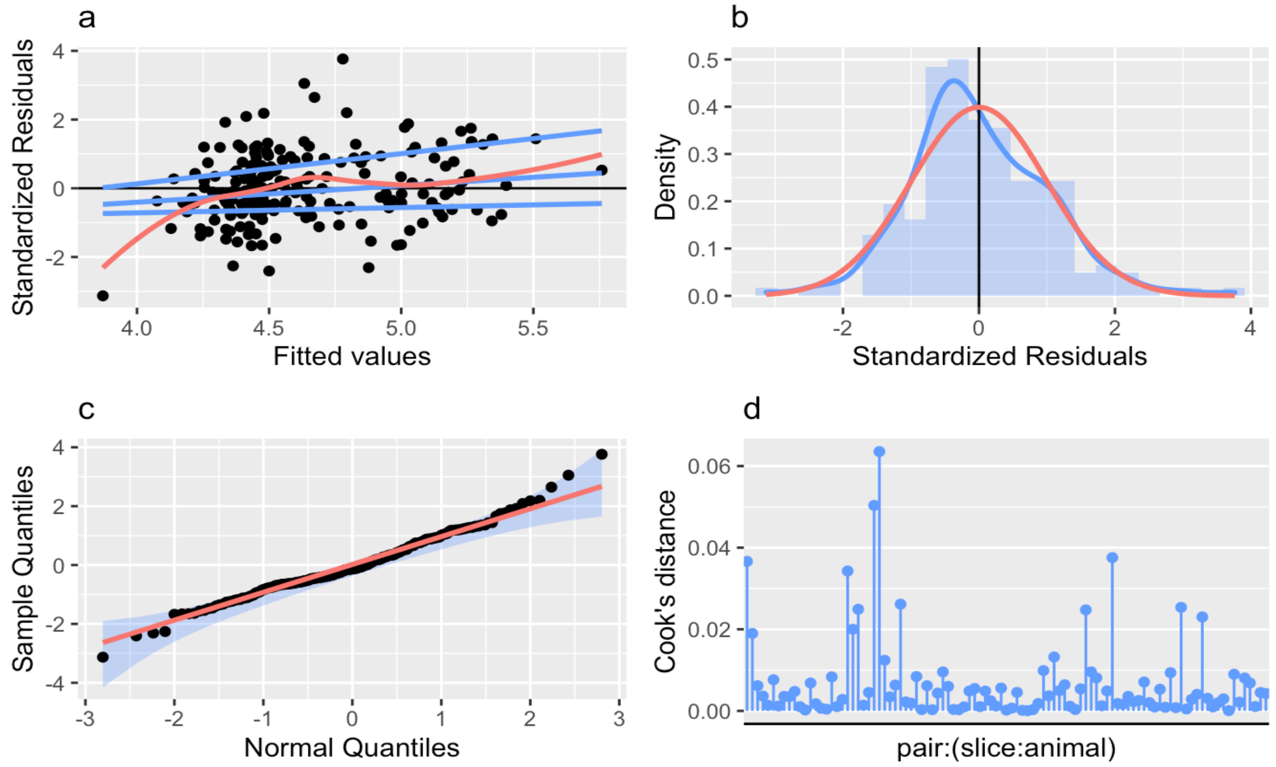

Figure S1.11. NMDA-EPSC risetimes in neurons expressing GluN2A knockout alleles.

Top. Table summary of a LMM predicting NMDA-EPSC 20-80% risetime from the transfection of Cre-GFP in CA1 neurons of *grin2a*$^{fl/fl}$ neurons. Fixed effects are summarised as an ANOVA table with the interaction term split into polynomial contrasts and followed up with posthoc pairwise comparisons. The *p*-value and Bayes factor for the interaction term omnibus test indicate a highly significant effect of loss-of-function (null) GluN2A alleles on the risetime of NMDA-EPSCs. Of the variance not accounted for by the fixed effects, 4%, 49%, 15% and 32% was explained by variability between recording pairs, slices, animals, and residual (unexplained) variance respectively. ***Bottom***. Model residuals appeared to be homoscedastic (**a**) and normally distributed (**b**, **c**), without outliers (**a, c**) or influential data points (**d**). A breakdown of samples sizes is reported in Table S4.

**Response**					
NMDA-EPSC 20-80% risetime (rise)					
**Formula**					
$\log(\boldsymbol{rise}) \sim genotype * transfection + (1 \vert animal/slice/pair)$					
**Source (Fixed)**	***F***	***df***	***df***$_{res}$	***p***	***BF***$_{10}$
genotype	1.5	2	9.69		
transfection	83.79	1	96		
genotype:transfection	44.21	2	96	<.001	2.33E+13
Linear	81.48	1	96	<.001	
Quadratic	5.55	1	96	.021	
**Posthoc test**	***t***		***df***	p_{adj}	
+/+ vs. +/-	1.07		96	.03	
+/+ vs. -/-	1.3		96	<.001	
+/- vs. -/-	1.22		96	<.001	
**Source (random)**	***N***	**ICC**			
pair:(slice:animal)	125	.04			
slice:animal	78	.49			
animal	27	.15			
Residual		.32			

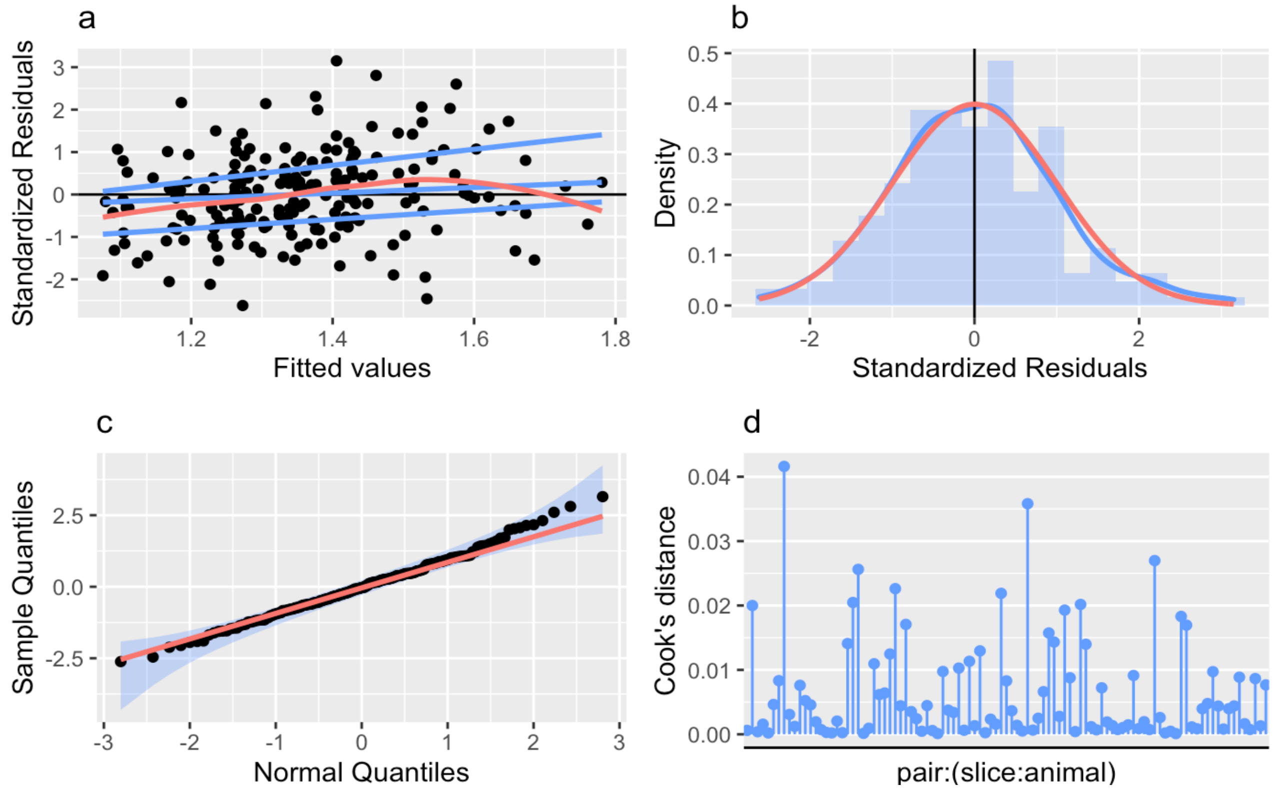

Figure S2: Likely toxicity from overexpressing the GluN2A L812M mutant

Example low magnification images and quantification of transfected cell density for GluN2A WT or L812M overexpressed in cultured hippocampal neurons. NMDA receptor blocker memantine (Mem) was effective at rescuing the density of neurons transfected with GluN2A L812M. The data points are plotted together with a bar graph representing the geometric mean and 95% confidence intervals.

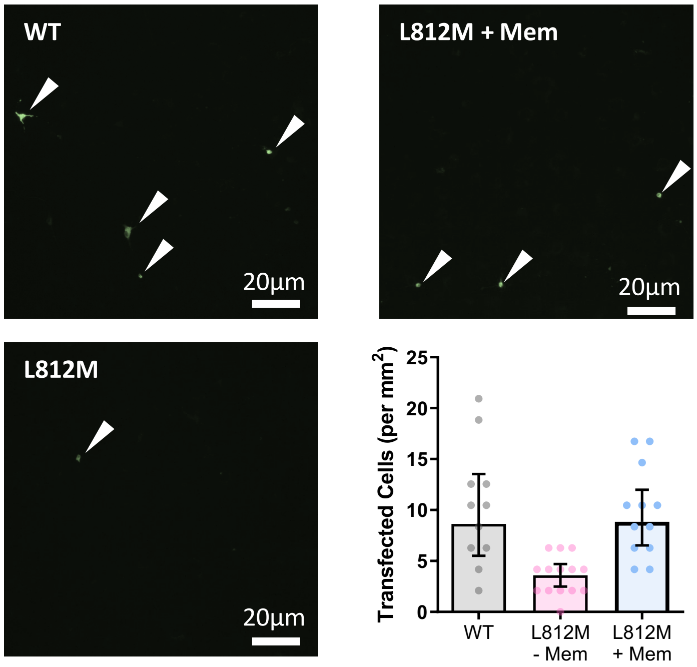

Figure S3: Effective rescue of native mouse GluN2A by human GluN2A

a) The experimental approach used to investigate the effect of missense mutations, by co-expressing Cre-GFP with human *GRIN2A* cDNA by single-cell electroporation around day 7 *in vitro*. The action of Cre recombinase results in the decline of native mouse GluN2A expression in Cre-transfected neurons of organotypic slices from *grin2a*$^{fl/fl}$ (as well as GluN2B in *grin2a*$^{fl/fl}$ *b*$^{fl/fl}$) mice slices. Meanwhile, native GluN2A is replaced by the co-expressed human GluN2A.
b) The transfected/untransfected ratios for NMDAR-EPSC peak amplitude and weighted decay time constants for varying amounts of co-transfected human *GRIN2A* cDNA. Arrow indicates the concentration that effectively rescued the peak amplitude and decay time constant of NMDAR-EPSCs. Error bars are 95% CI. The grey arrow indicates the concentration of *GRIN2A* cDNA used to rescue NMDA-EPSCs in experiments relating to Figures 2c, 2d, 4 and S3. The number of cell pairs recorded were 9, 14, 15, 15, 16 and 16 for 1.1, 3.3, 3.8, 4.4, 5.6 and 10.9 ng/μL respectively.
c) Example traces of NMDA-EPSCs measured at +20 mV to illustrate rescue of peak amplitude (*top*) and peak-scaled to illustrate rescue of the decay kinetics (*bottom*), of GluN2AKO cells by human GluN2A.

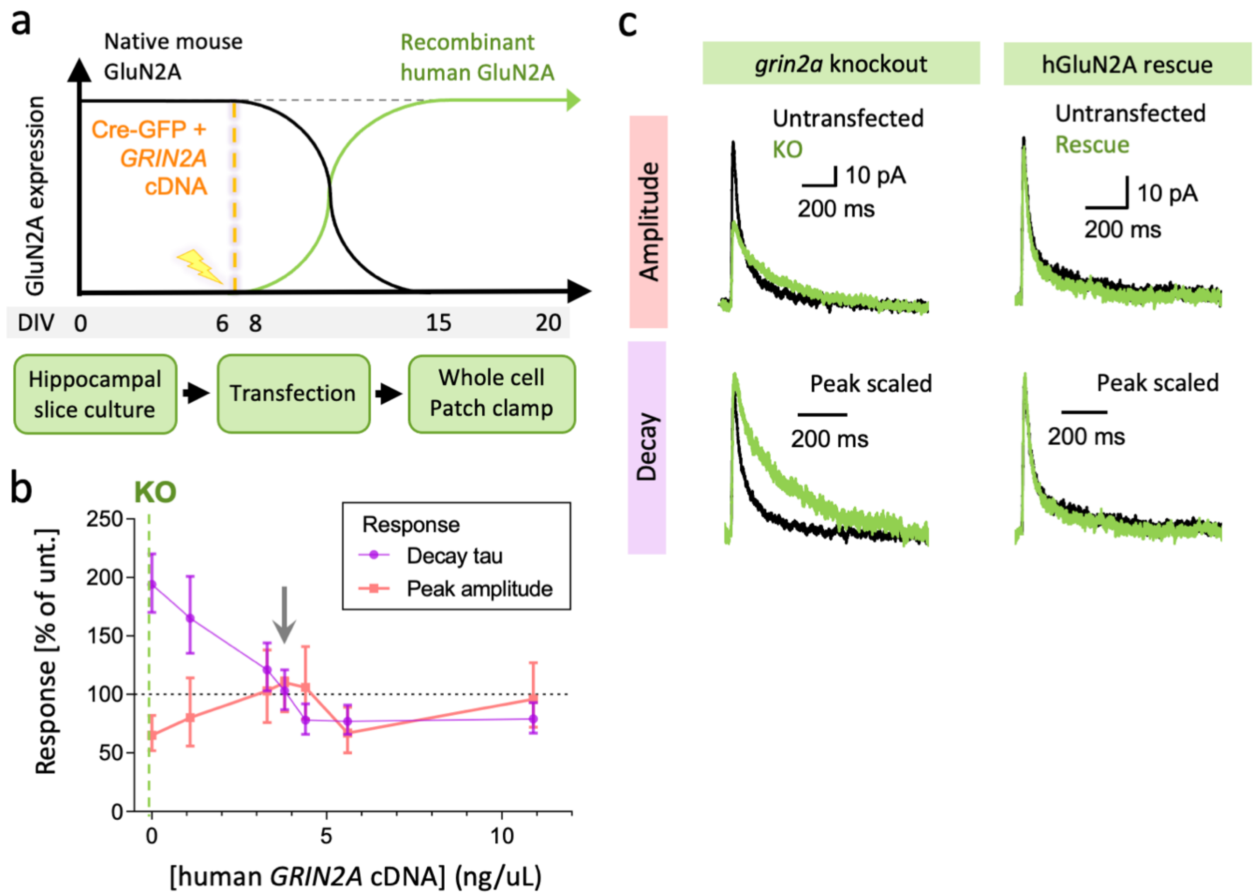

Figure S4: GluN2A mutations are not associated with large effects on AMPAR-EPSCs.

NMDA-EPSC$_{+20\ mV}$ peak amplitudes in *grin2a* $^{fl/fl}$ (untransfected) neurons and *grin2a* $^{-/-}$ neurons rescued with human GluN2A WT, LOF (C436R, T531M or R518H) or GOF (K669N or L812M) mutants (transfected). **ai)** Data points of measurements made in individual neurons. Matched data points, for simultaneously recorded untransfected and transfected neurons, are connected by a line. **aii)** Response ratios (transfected/untransfected) are expressed as a percentage and plotted for each pair of transfected-untransfected neurons. Crossbars in **i)** and **ii)** show the estimated marginal means with 95% confidence intervals backtransformed from the linear mixed models (Figure S11). Hypothesis tests are orthogonal contrasts based on *a priori* clustering of the mutations in Figure 1e. Standardized effect sizes (*r*) for comparisons of each mutant with WT for response ratios of peak amplitude in **aii** were .09, .07, .05, -.08 and -.08 (*N* = 125).

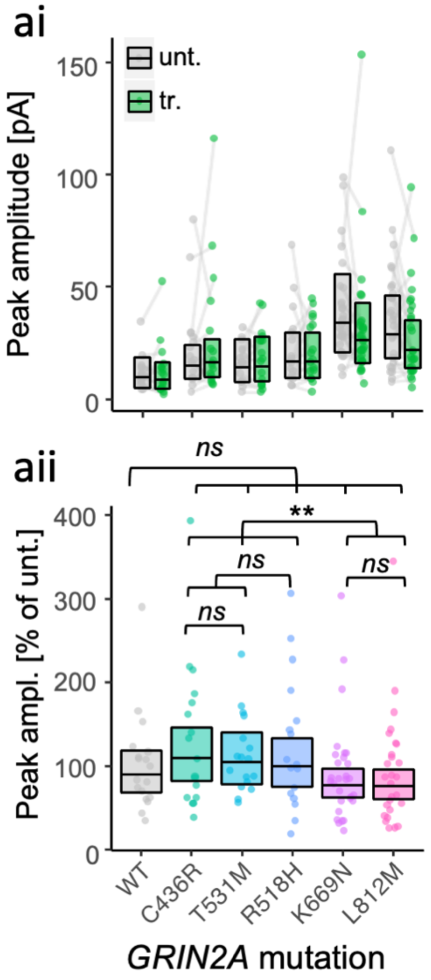

Figure S5: Structural models of the wildtype and mutated amino acids for LOF mutations

C436R disrupts a disulphide bridge. T531M modifies interactions around the LBD hinge between the upper and lower lobes of the clamshell. R518H disrupts key coulombic and hydrogen bonding interactions between the glutamate (Glu) ligand and the LBD.

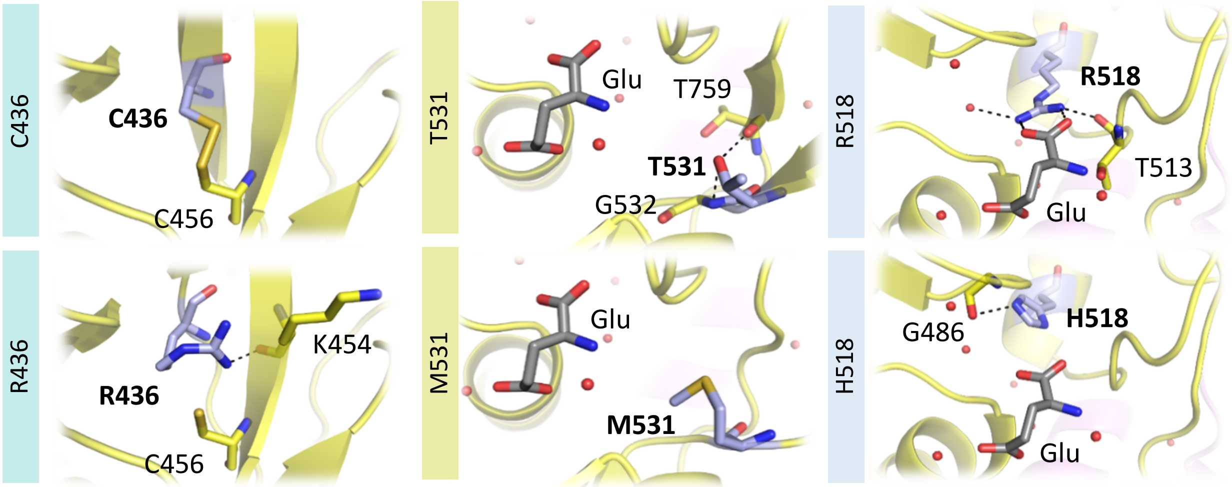

Table S1. Matrix of orthogonal contrasts based on *a priori* clustering of mutations.

Matrix of orthogonal contrasts used in the analysis of data in Figures 2ciii, 3bii, 4aiii, 4biii and 4ciii. Conditional table formatting is used to colour code the mutants involved in each contrast and illustrate their weighting.

Contrast	A	B	C	D	E
GRIN2A	**WT *vs* Mutants**	**LOF *vs* GOF**	**LOF2 *vs* LOF1**	**LOF2**	**GOF1**
**WT**	-0.83	0	0	0	0
**C436R**	0.17	-0.4	-0.33	-0.5	0
**T531M**	0.17	-0.4	-0.33	0.5	0
**R518H**	0.17	-0.4	0.67	0	0
**K669N**	0.17	0.6	0	0	-0.5
**L812M**	0.17	0.6	0	0	0.5

Table S2: Clinical features of patients reported previously with *GRIN2A* mutations used in this study.

GRIN2A mutation	Epilepsy Syndrome	Seizures	EEG	Speech	DD	ID	MRI	Ref
**C436R**	ABPE	Nocturnal GTCS, atonic seizures	CTS	Mild speech delay	Normal	Normal	ND	1
**T531M**	ECSWS, IEAD	Focal Dyscognitive seizure, Tonic Clonic Seizures	Intermittent discharges in clusters - LPH, HVBD (awake), Bilateral CTS (drowsiness & sleep), Multifocal discharges - mid parietal, independent bilateral bioccipital regions. Very frequent in awake state and continuous in sleep	Speech / Language Difficulties	Mild, Global	Mild, Learning difficulties	Normal	2
**R518H**	CSWSS, Landau-Kleffner Syndrome, atypical RE	Partial seizures	ESES, CTS	verbal dyspraxia	Normal	ND	Normal	3
**K669N**	CSWSS	Partial seizures	ESES	Language regression	Profound, Global	ND	Normal	3
**L812M**	Intractable infantile-onset epilepsy	Daily, Generalised seizures	Diffusely slow, disorganized background, irregularly generalized/lateralized spike and slow wave discharges, slow activity (3.5–4.5 Hz, 1–3 Hz delta and disorganized activity), intermittent irregular high amplitude 2.5 Hz spike-wave discharges (frontal, greater on right hemisphere).	ND	Profound, Global	Profound	Diffuse cerebral parenchymal volume loss, thin corpus callosum	4,5

ABPE - Atypical benign partial epilepsy, CSWSS - continuous spike-wave of slow sleep, CTS – centrotemporal spikes, DD – developmental delay, ECSWS - epileptic encephalopathy with continuous spike-and-wave during sleep syndrome, EEG – electroencephalogram, ESES - Electrical status epilepticus during slow-wave sleep, GTCS - generalized tonic-clonic seizure, HVBD - High voltage bilateral discharges, ID – intellectual disability, IEAD - intermediate epilepsy-aphasia disorder, LPH – Left posterior hemisphere, MRI – magnetic resonance imaging, ND – Not determined , RE – Rolandic Epilepsy

Table S3: Primers used to generate mutant GluN2A constructs from WT pCI-Neo *GRIN2A*

GRIN2A MUTATION	Forward primer (5' to 3')	Reverse primer (5' to 3')
**C436R**	AGGAACACCGTGCCACGTCGGAAGTTCGTCA	TGACGAACTTCCGACGTGGCACGGTGTTCCT
**R518H**	CCATCAATGAGGAACATTCTGAAGTGGTGGA	TCCACCACTTCAGAATGTTCCTCATTGATGG
**T531M**	TGCCCTTTGTGGAAATGGGAATCAGTGTCAT	ATGACACTGATTCCCATTTCCACAAAGGGCA
**K669N**	CCTCAGTGACAAAAATTTTCAGAGACCTCAT	ATGAGGTCTCTGAAAATTTTTGTCACTGAGG
**L812M**	GTGATGAGCAGCCAGATGGACATTGACAACA	TGTTGTCAATGTCCATCTGGCTGCTCATCAC

Table S4. Experiment sample sizes

Sample sizes for experiments detailed in Figures S1.1-1.11 broken down into the number of recording (cell) pairs, number of slices and number of animals.

Experiment relating to Figure 2b			
**Condition**	**pairs**	**slices**	**animals**
DKO	19	11	3
DKO DQP	15	9	3

Experiment relating to Figure 2c, d			
Mutation	pairs	slices	animals
WT	14	8	3
C436R	19	12	3
T531M	9	5	2
R518H	13	8	2
K669N	21	10	3
L812M	17	11	3
WT(2B)	19	13	3

Experiment relating to Figure 3b		
Mutation	roi	expt
WT	57	6
C436R	20	3
T531M	41	3
R518H	33	3
K669N	22	3
L812M	18	3
NONE	81	7

Experiment relating to Figure 4			
Mutation	pairs	slices	animals
WT	18	12	3
C436R	17	12	6
T531M	17	9	3
R518H	17	11	4
K669N	29	15	5
L812M	27	19	6

Experiment relating to Figure 5			
Genotype	pairs	slices	animals
WT	33	19	4
HET	29	15	6
HOM	37	19	4

Supplemental References

1. Lemke, J. R. *et al.* Mutations in GRIN2A cause idiopathic focal epilepsy with rolandic spikes. *Nat. Genet.* **45**, 1067–1072 (2013).
2. Carvill, G. L. *et al.* GRIN2A mutations cause epilepsy-aphasia spectrum disorders. *Nat. Genet.* **45**, 1073–1076 (2013).
3. Lesca, G. *et al.* GRIN2A mutations in acquired epileptic aphasia and related childhood focal epilepsies and encephalopathies with speech and language dysfunction. *Nat. Genet.* **45**, 1061–1066 (2013).
4. Pierson, T. M. *et al.* GRIN2A mutation and early-onset epileptic encephalopathy: personalized therapy with memantine. *Ann. Clin. Transl. Neurol.* **1**, 190–198 (2014).
5. Yuan, H. *et al.* Functional analysis of a de novo GRIN2A missense mutation associated with early-onset epileptic encephalopathy. *Nat. Commun.* **5**, 3251 (2014).
