# Supplementary material for: Common synaptic phenotypes arising from diverse mutations in the human NMDA receptor subunit GluN2A
Source: Commun Biol. 2022 Feb 28;5:174. doi: 10.1038/s42003-022-03115-3 (PMC8885697; doi:10.1038/s42003-022-03115-3)
Supplement: Supplementary file 3 — Description of Additional Supplementary Files [file 42003_2022_3115_MOESM3_ESM.pdf]

# Description of Additional Supplementary Files

**File name:** Supplementary Data 1
**Description:** Source data for Figure 1, including raw and normalized functional data for the various mutations, EFA loadings, and EFA scores.

**File name:** Supplementary Data 2
**Description:** Source data for Figure 2 (double knock-out mutants).

**File name:** Supplementary Data 3
**Description:** Source data Figure 3b.

**File name:** Supplementary Data 4
**Description:** Source data for Figure 4.

**File name:** Supplementary Data 5
**Description:** Source data for Figure 5.

**File name:** Supplementary Data 6
**Description:** Source data for Figure 6ci and Figure 6ei.
