# Supplementary material for: Common synaptic phenotypes arising from diverse mutations in the human NMDA receptor subunit GluN2A
Source: Commun Biol. 2022 Feb 28;5:174. doi: 10.1038/s42003-022-03115-3 (PMC8885697; doi:10.1038/s42003-022-03115-3)
Supplement: Supplementary file 10 — Reporting Summary [file 42003_2022_3115_MOESM10_ESM.pdf]

nature research

Corresponding author(s): Andrew Penn

Last updated by author(s): Feb 7, 2022

Reporting Summary

Nature Research wishes to improve the reproducibility of the work that we publish. This form provides structure for consistency and transparency in reporting. For further information on Nature Research policies, see our Editorial Policies and the Editorial Policy Checklist.

Statistics

For all statistical analyses, confirm that the following items are present in the figure legend, table legend, main text, or Methods section.

n/a	Confirmed	
☐	☒	The exact sample size (n) for each experimental group/condition, given as a discrete number and unit of measurement
☐	☒	A statement on whether measurements were taken from distinct samples or whether the same sample was measured repeatedly
☐	☒	The statistical test(s) used AND whether they are one- or two-sided<br>*Only common tests should be described solely by name; describe more complex techniques in the Methods section.*
☐	☒	A description of all covariates tested
☐	☒	A description of any assumptions or corrections, such as tests of normality and adjustment for multiple comparisons
☐	☒	A full description of the statistical parameters including central tendency (e.g. means) or other basic estimates (e.g. regression coefficient) AND variation (e.g. standard deviation) or associated estimates of uncertainty (e.g. confidence intervals)
☐	☒	For null hypothesis testing, the test statistic (e.g. F, t, r) with confidence intervals, effect sizes, degrees of freedom and P value noted<br>*Give P values as exact values whenever suitable.*
☐	☒	For Bayesian analysis, information on the choice of priors and Markov chain Monte Carlo settings
☐	☒	For hierarchical and complex designs, identification of the appropriate level for tests and full reporting of outcomes
☐	☒	Estimates of effect sizes (e.g. Cohen's d, Pearson's r), indicating how they were calculated

Our web collection on statistics for biologists contains articles on many of the points above.

Software and code

Policy information about availability of computer code

Data collection	Electrophysiology data was acquired using open source ACQ4 (v0.9.3)<br>Imaging data was acquired using commercial GRYPHAX software (v2.2.0, Jenoptik).
Data analysis	Images were analysed using open source FIJI software (v1.53) supplemented with custom macros and plugins (available at https://github.com/acp29/pimage/)<br>Electrophysiology traces were analysed in Stimfit software (v0.13 or 0.15.8) along with custom functions in Python (https://github.com/acp29/penn/) or Matlab R2017a+ (https://github.com/acp29/Elmasri_GRIN2A).<br>Statistical analysis was carried out in R (v4.1.0) and RStudo (v1.4). Rmarkdown code and knitted output is available at https://github.com/acp29/Elmasri_GRIN2A.<br>Confidence intervals and p-value for Kendall's tau correlation coefficient were determined by bootstrap using open source software iboot (part of Octave's statistics-bootstrap package, https://gnu-octave.github.io/packages/statistics-bootstrap)

For manuscripts utilizing custom algorithms or software that are central to the research but not yet described in published literature, software must be made available to editors and reviewers. We strongly encourage code deposition in a community repository (e.g. GitHub). See the Nature Research guidelines for submitting code & software for further information.

Data

Policy information about availability of data

All manuscripts must include a data availability statement. This statement should provide the following information, where applicable:

- Accession codes, unique identifiers, or web links for publicly available datasets
- A list of figures that have associated raw data
- A description of any restrictions on data availability

The data and scripts used for statistical analysis are available at https://github.com/acp29/Elmasri_GRIN2A and the linked RPubs pages

Field-specific reporting

Please select the one below that is the best fit for your research. If you are not sure, read the appropriate sections before making your selection.

☒ Life sciences ☐ Behavioural & social sciences ☐ Ecological, evolutionary & environmental sciences

For a reference copy of the document with all sections, see nature.com/documents/nr-reporting-summary-flat.pdf

Life sciences study design

All studies must disclose on these points even when the disclosure is negative.

Sample size	Data was collected until the number of cell pairs reached between a total of about 15-30 per mutation (or genotype)
Data exclusions	Incomplete data was excluded (e.g. because cell died before recording terminated). Pairs of neurons were discarded during data collection if the difference in series resistance varied by more than ~8 MΩ. All response measures associated with cell pairs that were identified as being influential outliers by multivariate outlier detection were excluded prior to statistical hypothesis testing.
Replication	The experiment was repeated in 3-6 animals.
Randomization	The choice of which slices/animals to allocate to which mutation or genotype was random.
Blinding	The experimenter was blind to the mutation or genotype of the sample.

Reporting for specific materials, systems and methods

We require information from authors about some types of materials, experimental systems and methods used in many studies. Here, indicate whether each material, system or method listed is relevant to your study. If you are not sure if a list item applies to your research, read the appropriate section before selecting a response.

Materials & experimental systems

n/a	Involved in the study	
☒	☐	Antibodies
☒	☐	Eukaryotic cell lines
☒	☐	Palaeontology and archaeology
☐	☒	Animals and other organisms
☒	☐	Human research participants
☒	☐	Clinical data
☒	☐	Dual use research of concern

Methods

n/a	Involved in the study	
☒	☐	ChIP-seq
☒	☐	Flow cytometry
☒	☐	MRI-based neuroimaging

Animals and other organisms

Policy information about studies involving animals; ARRIVE guidelines recommended for reporting animal research

Laboratory animals	Mice (C57Bl6 background): grin2a[fl/fl], grin2b[fl/fl], and grin2a[fl/fl] grin2b[fl/fl]. Neonatal mice from both sexes were used to create organotypic hippocampal slices. Dissociated cultures of neurons were obtained from Sprague-Dawley rats.
Wild animals	none
Field-collected samples	none
Ethics oversight	Schedule 1 training of the experimenters was overseen and approved by the local Named Training and Competency Officer (NTCO).

Ethics oversight	Experiments were reviewed a priori by the Animal Welfare Ethical Review Body at the University of Sussex and the project was licenced by the Home Office (UK, PPL# P6CF775B9).

Note that full information on the approval of the study protocol must also be provided in the manuscript.
